# Synthesis, characterisation and Pickering emulsifier performance of poly(stearyl methacrylate)–poly(*N*-2-(methacryloyloxy)ethyl pyrrolidone) diblock copolymer nano-objects *via* RAFT dispersion polymerisation in *n*-dodecane†
†Electronic supplementary information (ESI) available: PSMA_14_–PBzMA_95_ experimental details, assigned NMR spectra, analysis of PSMA_14_–PNMEP_*x*_ diblocks prepared at 20% w/w solids and further Pickering emulsion data. See DOI: 10.1039/c6py00138f



**DOI:** 10.1039/c6py00138f

**Published:** 2016-02-18

**Authors:** V. J. Cunningham, S. P. Armes, O. M. Musa

**Affiliations:** a Department of Chemistry , University of Sheffield , Brook Hill , Sheffield , South Yorkshire S3 7HF , UK . Email: s.p.armes@sheffield.ac.uk; b Ashland Specialty Ingredients , 1005 US 202/206 , Bridgewater , NJ 08807 , USA

## Abstract

A near-monodisperse poly(stearyl methacrylate) macromolecular chain transfer agent (PSMA macro-CTA) was prepared *via* reversible addition–fragmentation chain transfer (RAFT) solution polymerisation in toluene. This PSMA macro-CTA was then utilised as a stabiliser block for the RAFT dispersion polymerisation of a highly polar monomer, *N*-2-(methacryloyloxy)ethyl pyrrolidone (NMEP), in *n*-dodecane at 90 °C. ^1^H NMR studies confirmed that the rate of NMEP polymerisation was significantly faster than that of a non-polar monomer (benzyl methacrylate, BzMA) under the same conditions. For example, when targeting a PSMA_14_–PNMEP_100_ diblock copolymer, more than 99% NMEP conversion was achieved within 30 min, whereas only 19% BzMA conversion was obtained on the same time scale for the corresponding PSMA_14_–PBzMA_100_ synthesis. The resulting PSMA–PNMEP diblock copolymer chains underwent polymerisation-induced self-assembly (PISA) during growth of the insoluble PNMEP block to form either spherical micelles, highly anisotropic worms or polydisperse vesicles, depending on the target DP of the PNMEP chains. Systematic variation of this latter parameter, along with the solids content, allowed the construction of a phase diagram which enabled pure morphologies to be reproducibly targeted. Syntheses conducted at 10% w/w solids led to the formation of kinetically-trapped spheres. A monotonic increase in particle diameter with PNMEP DP was observed for such PISA syntheses, with particle diameters of up to 462 nm being obtained for PSMA_14_–PNMEP_960_. Increasing the copolymer concentration to 15% w/w solids led to worm-like micelles, while vesicles were obtained at 27.5% w/w solids. High (≥95%) NMEP conversions were achieved in all cases and 3 : 1 chloroform/methanol GPC analysis indicated relatively high blocking efficiencies. However, relatively broad molecular weight distributions (*M*_w_/*M*_n_ > 1.50) were observed when targeting PNMEP DPs greater than 150. This indicates light branching caused by the presence of a low level of dimethacrylate impurity. Finally, PSMA_14_–PNMEP_49_ spheres were evaluated as Pickering emulsifiers. Unexpectedly, it was found that either water-in-oil or oil-in-water Pickering emulsions could be obtained depending on the shear rate employed for homogenisation. Further investigation suggested that high shear rates lead to *in situ* inversion of the initial hydrophobic PSMA_14_–PNMEP_49_ spheres to form hydrophilic PNMEP_49_–PSMA_14_ spheres.

## Abstract 2

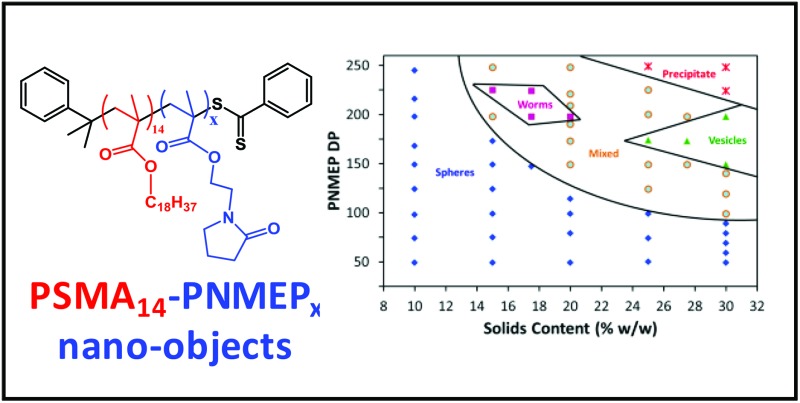
Block copolymer spheres, worms or vesicles can be prepared *via* RAFT dispersion polymerisation of *N*-(methacryloyloxy)ethyl pyrrolidone in *n*-dodecane using a poly(stearyl methacrylate) chain transfer agent.

## Introduction

It is well-known that AB diblock copolymers undergo self-assembly both in the solid state and also in solution.^1–3^ In the latter case, a diverse range of copolymer morphologies has been reported, including spheres,^4^ worms^5,6^ or vesicles.^7^ Typically, the copolymer chains are first prepared in a non-selective solvent and then subjected to either a gradual change in solvency or a pH switch in a separate step, which is typically undertaken in dilute solution.

In recent years, polymerisation-induced self-assembly (PISA) of diblock copolymers in a solvent that is selective for the growing second block has become increasingly popular.^8–10^ PISA offers two decisive advantages over traditional processing methods:^11^ (i) syntheses can be conducted at up to 50% w/w solids^12,13^ and (ii) diblock copolymer nanoparticles are obtained directly, without requiring any post-polymerisation processing steps. When combined with PISA, controlled radical polymerisation techniques such as atom transfer radical polymerisation (ATRP)^14,15^ or reversible addition–fragmentation chain transfer (RAFT) polymerisation^16–19^ have enabled the preparation of a wide range of well-defined nanoparticles.^10,20,21^ In particular, RAFT dispersion polymerisation allows the efficient synthesis of pure spherical, worm-like or vesicular morphologies in aqueous,^22–25^ alcoholic^26–29^ or non-polar media^13,30–36^ as well as ionic liquids.^37^

Of particular relevance to the present work is the RAFT-mediated synthesis of well-defined poly(lauryl methacrylate)–poly(benzyl methacrylate) (PLMA–PBzMA) diblock copolymer nanoparticles in *n*-alkanes.^33^ Fielding *et al.* reported that using a relatively long PLMA macromolecular chain transfer agent (macro-CTA) only led to spherical nanoparticles regardless of the target PBzMA DP, whereas using a relatively short PLMA macro-CTA enabled the production of spherical, worm-like or vesicular nanoparticles in *n*-heptane. Switching the solvent to *n*-dodecane allowed a detailed study of the thermo-responsive behaviour of PLMA_16_–PBzMA_37_ diblock copolymer worms.^34^ Heating from 20 °C to 90 °C led to a worm-to-sphere order–order transition as a result of surface plasticisation of the worm cores by the hot solvent, which causes a subtle change in the packing parameter, *P*.^38^ More recently, Derry and co-workers used a similar PLMA–PBzMA formulation to target spherical nanoparticles *via* a highly convenient one-pot protocol in industrially-relevant solvents such as mineral oil or a poly(α-olefin) at up to 50% w/w solids.^13^

**Scheme 1:**
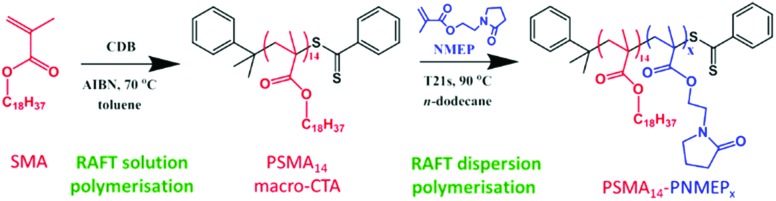
Synthesis of a PSMA_14_ macro-CTA by RAFT solution polymerisation of SMA followed by the preparation of PSMA_14_–PNMEP_*x*_ nano-objects *via* RAFT dispersion polymerisation of NMEP in *n*-dodecane at 90 °C.

In the present work, we describe the synthesis of a range of new poly(stearyl methacrylate)–poly(*N*-2-(methacryloyloxy)ethyl pyrrolidone) (PSMA–PNMEP) diblock copolymer nano-objects *via* RAFT dispersion polymerisation of NMEP in *n*-dodecane, see Scheme 1. The diblock copolymer chains are characterised by ^1^H NMR and gel permeation chromatography (GPC), while dynamic light scattering (DLS) and transmission electron microscopy (TEM) have been used to assess the particle size and morphology. A phase diagram has been constructed to enable pure spherical micelles, worm-like micelles or vesicles to be reproducibly targeted. In addition, these PSMA-PNMEP spheres have been evaluated as putative Pickering emulsifiers.

## Experimental

### Materials

Stearyl methacrylate (SMA), cumyl dithiobenzoate (CDB) and *n*-dodecane were purchased from Sigma-Aldrich (UK) and were used as received. *N*-2-(Methacryloyloxy)ethyl pyrrolidone (NMEP, 96% or 98% purity) was donated by Ashland Specialty Ingredients (USA) and was used without further purification. Azoisobutyronitrile (AIBN) was purchased from Molekula (Dorset, UK). *tert*-Butyl peroxy-2-ethylhexanoate (T21s) was purchased from AkzoNobel (The Netherlands). CD_2_Cl_2_ was purchased from Goss Scientific Ltd, (UK) and CDCl_3_ was purchased from VWR chemicals (UK). All other solvents were purchased from Fisher Scientific (Loughborough, UK) and were used as received.

### Preparation of PSMA_14_ macro-CTA

SMA (33.4765 g, 0.099 mol), CDB RAFT agent (5.1690 g, 19 mmol; target degree of polymerisation, DP = 5) and AIBN (0.6233 g, 3.8 mmol; CTA/initiator molar ratio = 5.0) were weighed into a 250 ml round-bottomed flask. Toluene (58 ml) was deoxygenated separately with nitrogen for 30 min prior to addition to the other reagents. The reaction solution was stirred and degassed in an ice bath for a further 30 min, before placing in an oil bath at 70 °C. The polymerisation was allowed to proceed for 10 h, resulting in a final monomer conversion of 80% as judged by ^1^H NMR. The crude homopolymer was purified by precipitating into a ten-fold excess of ethanol. This purification step was repeated twice to afford a pure PSMA macro-CTA (21.6 g, <1% residual monomer). The mean degree of polymerisation was calculated to be 14, as judged by ^1^H NMR spectroscopy by comparing the integrated aromatic CDB proton signals at 7.0–8.0 ppm with that assigned to the two oxymethylene PSMA protons at 3.6–4.2 ppm. GPC analysis using a 3 : 1 v/v chloroform/methanol mixed eluent indicated an *M*_n_ of 7500 g mol^–1^ and an *M*_w_/*M*_n_ of 1.12 (*vs.* a series of near-monodisperse poly(methyl methacrylate) calibration standards).

### Synthesis of PSMA_14_–PNMEP_*x*_ *via* RAFT dispersion polymerisation of SMA

A typical protocol for the synthesis of PSMA_14_–PNMEP_98_ diblock copolymer nanoparticles was as follows: PSMA_14_ macro-CTA (0.0706 g), NMEP (0.2787 g, 1.413 mmol; target DP = 100), T21s (0.755 mg, 3.49 μmol; dissolved at 10% v/v in *n*-dodecane; CTA/T21s molar ratio = 4.0) were dissolved in *n*-dodecane (4.1 ml, 10% w/w) in a 25 ml round-bottomed flask. The reaction mixture was sealed and purged with nitrogen for 30 min, prior to immersion in an oil bath set at 90 °C for 2 h. The resulting copolymer was analysed by GPC using a 3 : 1 chloroform/methanol mixed eluent (*M*_n_ = 49 600 g mol^–1^, *M*_w_/*M*_n_ = 1.19 *vs.* PMMA standards). ^1^H NMR spectroscopy analysis of the final reaction solution diluted approximately ten-fold in CDCl_3_ indicated 98% NMEP conversion. DLS studies conducted on a 0.20% w/w copolymer dispersion indicated an intensity-average particle diameter of 36 nm (DLS polydispersity, PDI = 0.01). Other diblock copolymer compositions were targeted by adjusting the NMEP/PSMA_14_ macro-CTA molar ratio and/or by varying the volume of solvent in the PISA formulation.

### Preparation of Pickering emulsions using PSMA_14_–PNMEP_49_ spherical nanoparticles

Water (2.0 ml) was homogenized with 2.0 ml of a 0.0675–2.50% w/w PSMA_14_–PNMEP_49_ diblock copolymer dispersion in *n*-dodecane for 2 min at 20 °C using an IKA Ultra-Turrax T-18 homogeniser equipped with a 10 mm dispersing tool. The shear rate was systematically varied between 3500 rpm and 24 000 rpm.

### Copolymer characterisation

#### ^1^H NMR spectroscopy

All ^1^H NMR spectra were recorded at 20 °C in either CD_2_Cl_2_ or CDCl_3_ using a 400 MHz Bruker Avance-400 spectrometer with 64 scans being averaged per spectrum.

#### Gel permeation chromatography (GPC)

The molecular weights and polydispersities of the PSMA_14_ macro-CTA and PSMA_14_–PNMEP_*x*_ diblock copolymers were obtained using a GPC set-up comprising a Hewlett Packard HP1090 Liquid Chromatograph pump unit and two Polymer Laboratories PL gel 5 μm ‘Mixed C’ columns connected in series with a guard column at 40 °C connected to a Gilson Model 131 refractive index detector. The eluent was a 3 : 1 v/v% chloroform/methanol mixture containing 2 mM LiBr at a flow rate of 1.0 ml min^–1^. A series of near-monodisperse poly(methyl methacrylate) (PMMA) standards were used for calibration. Data analysis was carried out using Cirrus GPC software supplied by Agilent.

#### Dynamic light scattering (DLS)

The intensity-average hydrodynamic diameter of each batch of nanoparticles was determined at 25 °C using a Malvern Zetasizer NanoZS instrument at a scattering angle of 173°. Dilute dispersions (0.20% w/w) in *n*-heptane were analysed using quartz cuvettes and data were averaged over three consecutive runs.

#### Transmission electron microscopy (TEM)

Copper/palladium TEM grids (Agar Scientific, UK) were coated in-house to yield a thin film of amorphous carbon. Dilute dispersions (0.20% w/w in *n*-heptane, 10.0 μL) were placed on the carbon-coated grids and left for 30 min to allow solvent evaporation. The grids were exposed to ruthenium(viii) oxide vapour for 7 min at 20 °C prior to analysis. Imaging was performed using a Philips CM100 instrument operating at 100 kV and equipped with a Gatan 1 k CCD camera.

The ruthenium(viii) oxide was prepared as follows: ruthenium(iv) oxide (0.30 g) was added to water (50 g) to form a black slurry; addition of sodium periodate (2.0 g) with stirring produced a yellow solution of ruthenium(viii) oxide within 1 min.

#### Optical microscopy

Optical microscopy images of emulsion droplets were recorded using a Motic DMBA300 digital biological microscope equipped with a built-in camera and Motic Images Plus 2.0 ML software.

#### Laser diffraction

Emulsions were sized using a Malvern Mastersizer 2000 instrument equipped with a small volume Hydro 2000SM sample dispersion unit (*ca*. 50 ml), a HeNe laser operating at 633 nm, and a solid-state blue laser operating at 466 nm. The stirring rate was adjusted to 1000 rpm in order to avoid creaming of the emulsion during analysis. After each measurement, the cell was rinsed once with ethanol, followed by two rinses with distilled water; the glass walls of the cell were carefully wiped with tissue to avoid cross-contamination and the laser was aligned centrally to the detector prior to data acquisition.

## Results and discussion

### Synthesis of PSMA macro-CTA *via* RAFT solution polymerisation

A PSMA macro-CTA was synthesised *via* RAFT solution polymerisation of SMA in toluene at 70 °C using cumyl dithiobenzoate (CDB) as a chain transfer agent (Scheme 1). The reaction was allowed to proceed for 10 h before being quenched; ^1^H NMR spectroscopy indicated 80% conversion and a mean degree of polymerisation (DP) of 14 after purification. GPC analysis of the purified PSMA macro-CTA using a 3 : 1 v/v chloroform/methanol mixed eluent indicated a *M*_n_ of 7500 g mol^–1^ with an *M*_w_/*M*_n_ of 1.12, which suggested good control for this pseudo-living polymerisation. A self-blocking chain extension experiment with a second charge of SMA monomer was used to examine the chain-end fidelity of the PSMA_14_ macro-CTA. GPC analysis of the resulting PSMA_101_ homopolymer confirmed a high blocking efficiency for the PSMA_14_ macro-CTA (see Fig. S1 in the ESI†), which indicated high RAFT chain-end fidelity.

### Kinetics of the RAFT dispersion polymerisation of NMEP targeting PSMA_14_–PNMEP_100_ at 20% w/w solids

**Fig. 1:**
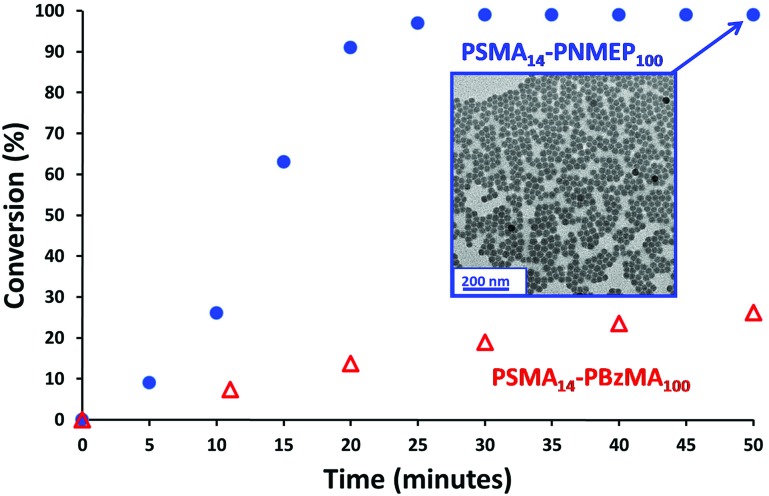
Kinetics of the polymerisation of NMEP and BzMA at 90 °C when targeting PSMA_14_–PNMEP_100_ (blue circles) and PSMA_14_–PBzMA_100_ (red triangles) at 20% w/w solids. Insert: transmission electron microscopy image obtained after 50 min for PSMA_14_–PNMEP_100_ showing the formation of near-monodisperse spherical nanoparticles with a mean diameter of 27 nm.

A kinetic study of the chain extension of the PSMA_14_ macro-CTA *via* RAFT dispersion polymerisation of NMEP in *n*-dodecane at 90 °C was conducted using a macro-CTA/initiator molar ratio of 4.0 (Scheme 1). Targeting a composition of PSMA_14_–PNMEP_100_ at 20% w/w solids, aliquots of the reaction solution were extracted under nitrogen every 5 min for 50 min with ^1^H NMR spectroscopy being used to monitor the extent of polymerisation (see Fig. S2 in ESI† for assigned ^1^H NMR spectra). Fig. 1 shows the conversion *vs.* time curve.

Approximately 90% conversion was attained within 20 min, with 99% conversion being achieved within 30 min. This is significantly faster than other RAFT dispersion polymerisations that have been conducted in *n*-alkanes.^13,31,33–35^ For example, Fielding *et al.* reported that the polymerisation of benzyl methacrylate at 90 °C in *n*-heptane using a PLMA_17_ macro-CTA at 15% solids took 5 h to reach 95% conversion.^33^ Moreover, these PLMA–PBzMA diblock copolymers were prepared using a lower macro-CTA/initiator molar ratio of 2.0 compared to the value of 4.0 used for the PSMA–PNMEP diblock copolymer synthesis reported in the present study. In view of our unexpected observations, we conducted a kinetic study of the synthesis of PSMA_14_–PBzMA_100_ in *n*-dodecane under precisely the same conditions employed for PSMA_14_–PNMEP_100_ in order to enable a direct comparison to be made between these two PISA formulations. Both NMEP polymerisations were performed at 90 °C using a macro-CTA/initiator molar ratio of 4.0 at 20% w/w solids. The kinetic data obtained for PSMA_14_–PBzMA_100_ are also shown in Fig. 1. A BzMA conversion of just 19% was achieved within 30 min (although 95% conversion was eventually achieved after 6 h), which indicates a much slower rate of polymerisation than that of NMEP (see Fig. S3 in ESI†). This is attributed to the highly polar nature of the latter monomer: similar polarity effects for monomers and solvents have been reported in the literature.^39–41^ TEM analysis of the diluted PSMA_14_–PNMEP_100_ dispersion recorded after 50 min (>99% conversion) revealed near-monodisperse spherical nanoparticles with a mean diameter of 27 ± 3 nm (Fig. 1, inset). GPC analysis of aliquots taken during the kinetic run indicated a monotonic increase in number-average molecular weight with conversion, with a final *M*_n_ of 49 900 and a relatively low final *M*_w_/*M*_n_ of 1.19 (See Fig. S4 in ESI†).

### Synthesis of a series of PSMA_14_–PNMEP_*x*_ diblock copolymer spheres *via* RAFT dispersion polymerisation

**Fig. 2:**
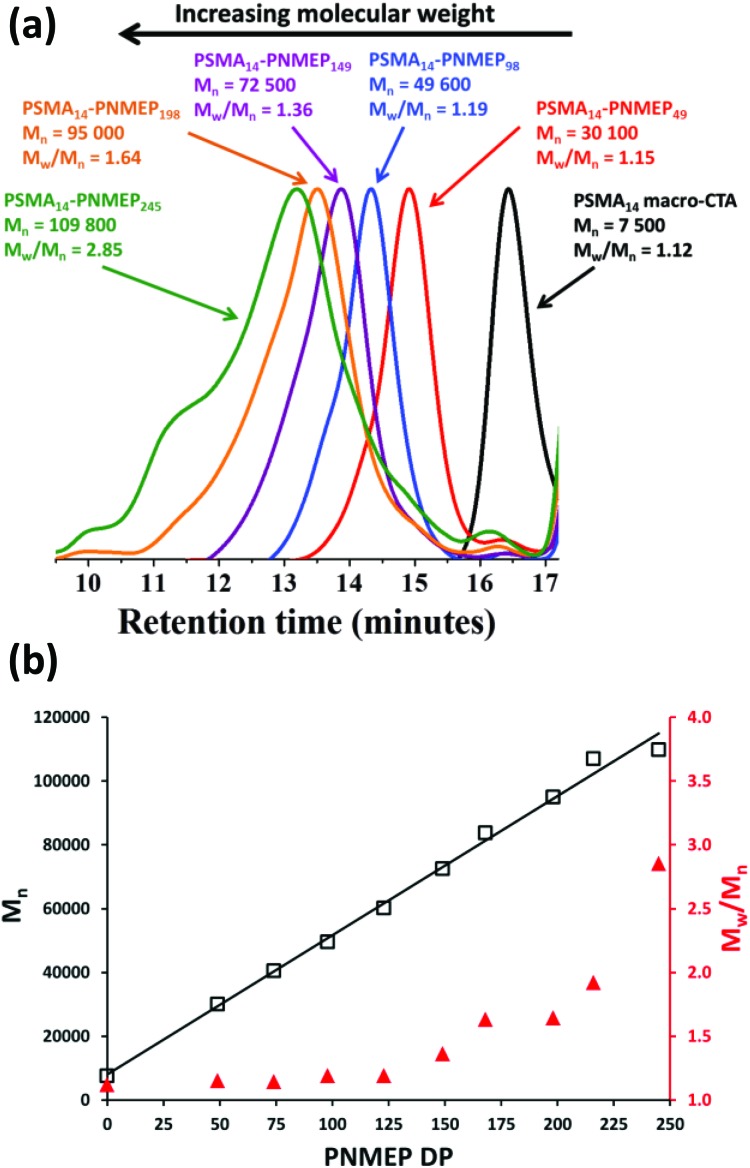
(a) 3 : 1 Chloroform/methanol GPC curves obtained for PSMA_14_–PNMEP_*x*_ diblock copolymer nanoparticles prepared at 10% w/w solids *via* RAFT dispersion polymerisation of NMEP at 90 °C. (b) Relationship between target PNMEP DP and GPC *M*_n_ (black squares) and *M*_w_/*M*_n_ (red triangles) for the same series of PSMA_14_–PNMEP_*x*_ diblock copolymer nanoparticles prepared at 10% w/w solids.

Utilising the above kinetic data, a series of PSMA_14_–PNMEP_*x*_ diblock copolymers were prepared at 10% w/w solids. The target degree of polymerisation (DP) for the PNMEP core-forming block (*x*) was systematically varied from 50 to 1000 (see Table 1) and relatively high (>96%) NMEP conversions were achieved in all cases. PSMA_14_–PNMEP_*x*_ diblock copolymers with a target PNMEP DP (*x*) of less than 250 were analysed by GPC. Representative chromatograms for *x* = 49, 98, 149, 198 and 245 are shown in Fig. 2a. All PSMA_14_–PNMEP_*x*_ diblock copolymers exhibited high blocking efficiencies relative to the PSMA_14_ macro-CTA and the copolymer *M*_n_ increased as higher PNMEP DPs were targeted, as expected. Fig. 2b shows the relationship between both *M*_n_ and *M*_w_/*M*_n_ with respect to the actual PNMEP DP, as calculated from the corresponding ^1^H NMR conversions assuming 100% blocking efficiency. A linear increase in *M*_n_ with PNMEP DP is observed, which is characteristic of a pseudo-living polymerisation. However, gradual broadening of the molecular weight distribution is also observed, with *M*_w_/*M*_n_ values reaching as high as 2.85 for PSMA_14_–PNMEP_245_. In principal, this progressive increase in *M*_w_/*M*_n_ when targeting higher PNMEP DPs could be the result of a dimethacrylate impurity in the NMEP monomer, which has a purity of only 96%. However, another plausible explanation could be chain transfer to polymer, with the two methylene carbonyl protons on the pyrrolidone ring being particularly prone to abstraction.^42^ Alternatively, the two pairs of azamethylene protons in the NMEP residues might participate in such a side reaction. When targeting DPs greater than 250, PSMA_14_–PNMEP_*x*_ diblock copolymers became insoluble in the 3 : 1 chloroform/methanol eluent and hence could not be analysed by GPC, suggesting that higher levels of cross-linking lead to a (micro)gel fraction. Fielding *et al.* also reported relatively high *M*_w_/*M*_n_ values for PLMA–PBzMA PISA formulations when targeting higher PBzMA DPs (PLMA_47_–PBzMA_900_, *M*_w_/*M*_n_ = 1.76).^33^ In contrast, Pei and co-workers obtained low-polydispersity poly(stearyl methacrylate)–poly(3-phenylpropyl methacrylate) (PSMA–PPPMA) diblock copolymers when using a slightly higher macro-CTA/initiator molar ratio of 5.0,^35^ although in this earlier study the target DP for the core-forming block was never higher than 165. In the present study, *M*_w_/*M*_n_ values only began to increase significantly for PSMA_14_–PNMEP_*x*_ when targeting *x* values greater than 150 (see Table 1).

**Table 1:** Conversions, molecular weights (*M*_n_), polydispersities (*M*_w_/*M*_n_) and mean DLS diameters obtained for PSMA_14_–PNMEP_*x*_ (or S_14_–N_*x*_) diblock copolymer nanoparticles prepared at various solids content and the corresponding PSMA_14_ macro-CTA

	Diblock composition	Conversion^*a*^ (%)	Solids content (% w/w)	GPC^*b*^		DLS particle diameter^*c*^ (nm)
				*M*_n_ (kg mol^–1^)	*M*_w_/*M*_n_	
1	S_14_	80	40	7.5	1.12	N/A
2	S_14_–N_49_	98	10	30.1	1.15	23 (0.205)
3	S_14_–N_74_	99	10	40.5	1.14	30 (0.028)
4	S_14_–N_98_	98	10	49.6	1.19	36 (0.035)
5	S_14_–N_124_	99	10	60.1	1.19	42 (0.034)
6	S_14_–N_149_	99	10	72.5	1.36	47 (0.054)
7	S_14_–N_168_	96	10	83.8	1.63	56 (0.008)
8	S_14_–N_198_	99	10	95.0	1.64	62 (0.015)
9	S_14_–N_216_	96	10	107.0	1.92	76 (0.025)
10	S_14_–N_245_	98	10	109.8	2.85	95 (0.005)
11	S_14_–N_270_	98	10	Not determined	Not determined	153 (0.006)
12	S_14_–N_291_	97	10	Not determined	Not determined	173 (0.006)
13	S_14_–N_392_	98	10	Not determined	Not determined	274 (0.028)
14	S_14_–N_485_	97	10	Not determined	Not determined	340 (0.035)
15	S_14_–N_960_	96	10	Not determined	Not determined	462 (0.010)

^*a*^Monomer conversion determined by ^1^H NMR spectroscopy in CDCl_3_.

^*b*^Determined by 3 : 1 v/v chloroform/methanol GPC against PMMA calibration standards using a refractive index detector.

^*c*^The number in brackets refers to the DLS polydispersity.

**Fig. 3:**
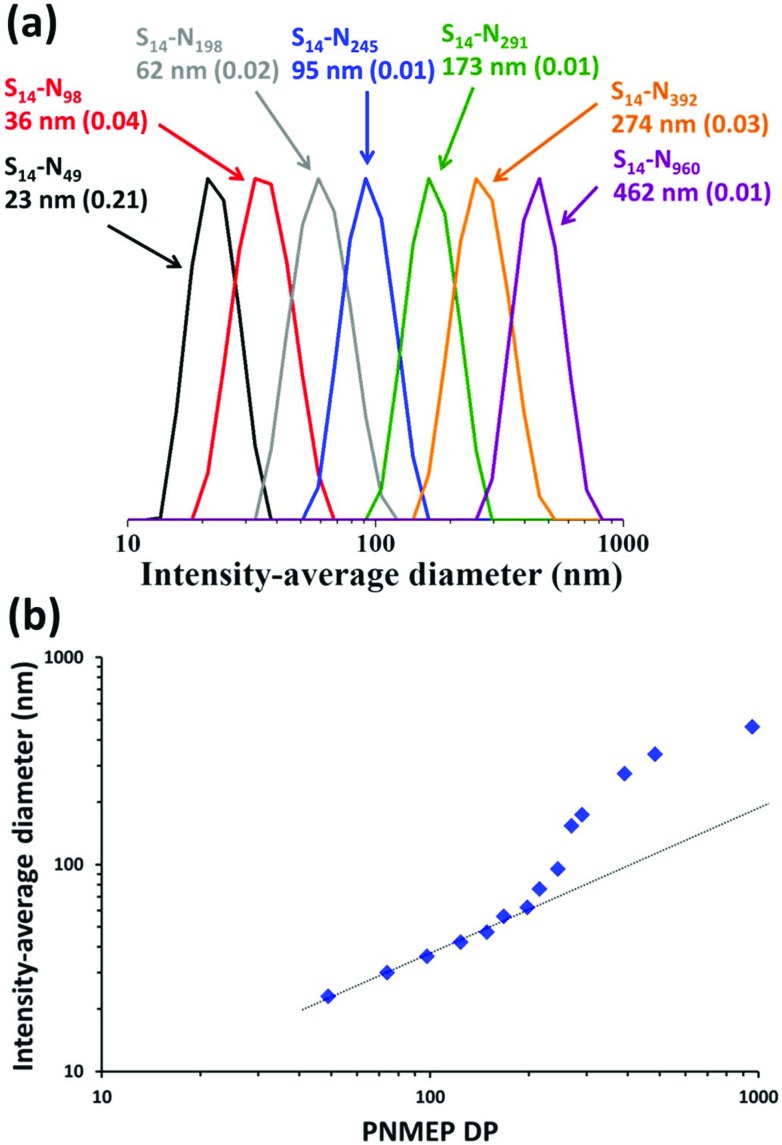
(a) DLS intensity-average size distributions for PSMA_14_–PNMEP_*x*_ diblock copolymer nanoparticles prepared *via* RAFT dispersion polymerisation of NMEP at 10% w/w solids in *n*-dodecane at 90 °C (N.B. for brevity ‘S’ denotes PSMA and ‘N’ denotes PNMEP, the numbers in brackets refer to the DLS polydispersity in each case). (b) A plot of intensity-average diameter *vs.* mean degree of polymerisation of the PNMEP core-forming block. TEM studies confirmed that spherical morphologies were obtained in all cases (see Fig. 4).

DLS analysis of these PSMA_14_–PNMEP_*x*_ diblock copolymer nanoparticles indicated a monotonic increase in the intensity-average diameter when targeting higher PNMEP DPs (Fig. 3a). DLS size distributions were relatively narrow in all cases: the smallest nanoparticles (PSMA_14_–PNMEP_49_) were only 23 nm in diameter, while the largest nanoparticles (PSMA_14_–PNMEP_960_) had a diameter of 462 nm. As far as we are aware, the latter particles are the largest spheres ever reported for PISA syntheses under any conditions.^12,43^ The relationship between DLS diameter and core-forming block DP is shown in Fig. 3b. There is an initial linear increase in particle size up to a DP of approximately 200, with a non-linear regime thereafter. This complex behaviour is not currently understood and clearly warrants further study.

**Fig. 4:**
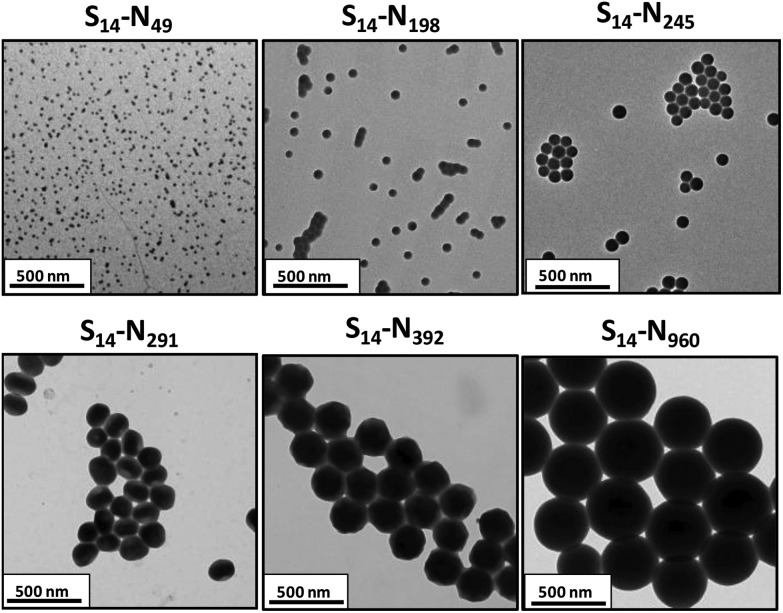
TEM images obtained for PSMA_14_–PNMEP_*x*_ diblock copolymer nanoparticles prepared at 10% w/w solids in *n*-dodecane showing well-defined spherical nanoparticles (N.B. for brevity ‘S’ denotes PSMA and ‘N’ denotes PNMEP).

TEM studies of the same series of PSMA_14_–PNMEP_*x*_ diblock copolymer nanoparticles prepared at 10% w/w solids indicated an exclusively spherical morphology, rather than higher order morphologies such as worms or vesicles (see Fig. 4). As for the DLS data, a monotonic increase in particle diameter is observed with increasing PNMEP DP. Eisenberg and co-workers have reported that, for post-polymerisation processing of polystyrene–poly(acrylic acid) diblock copolymers in dilute solution using a solvent switch, spherical nanoparticles can become kinetically trapped and hence no longer represent the equilibrium morphology.^11,44^ Similar effects have been observed for various PISA syntheses based on RAFT dispersion polymerisation.^23,27,33,45^ To examine whether this problem also applied to the current PISA formulation, a new series of PSMA_14_–PNMEP_*x*_ diblock copolymers were synthesised at 20% w/w solids. According to the PISA literature, such higher concentrations are often essential for accessing equilibrium non-spherical morphologies, *e.g.* worms or vesicles.^45^

### Construction of a PSMA_14_–PNMEP_*x*_ diblock copolymer phase diagram

The DP of the stabiliser block is an important parameter when targeting higher order morphologies. This is because a relatively high DP leads to more effective steric stabilisation during PISA, which in turn prevents the 1D sphere–sphere fusion that is the essential first step in the formation of worms.^22,33^ For example, Fielding *et al.* reported that a PLMA_37_ macro-CTA produced exclusively spherical nanoparticles, whereas using a shorter PLMA_17_ macro-CTA enabled the synthesis of worm-like micelles or vesicles.^33^ On this basis, the PSMA_14_ macro-CTA utilised herein was expected to be sufficiently short to stabilise the full range of morphologies at higher solids.

**Fig. 5:**
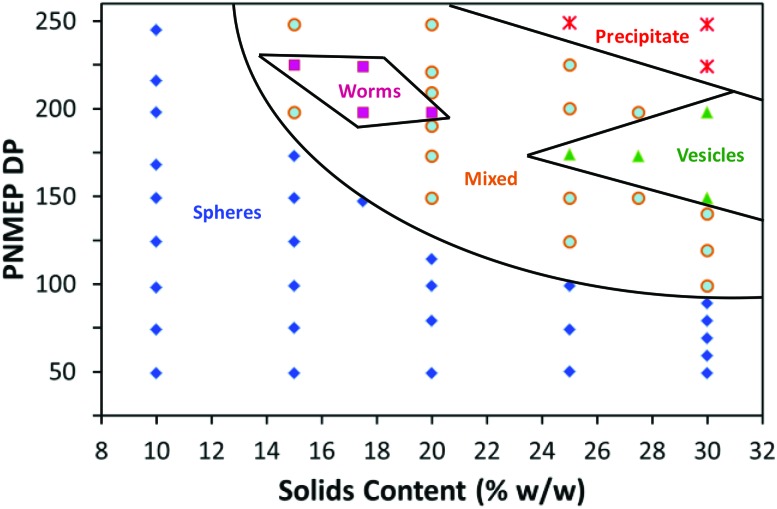
Phase diagram for a series of PSMA_14_–PNMEP_*x*_ diblock copolymers synthesised by RAFT dispersion polymerisation of NMEP in *n*-dodecane at various concentrations ranging between 10 and 30% w/w solids. Post-polymerisation analysis of each diblock copolymer dispersion by TEM determined the phase boundaries.

At least 95% conversion was achieved in all PSMA_14_–PNMEP_*x*_ syntheses conducted at 20% w/w solids (see Table S1†). GPC studies indicated an approximately linear increase in *M*_n_ with PNMEP DP between 49 and 248 (see Fig. S5 in ESI†). Like the GPC data obtained at 10% w/w solids, significantly broader molecular weight distributions were observed when targeting PSMA_14_–PNMEP_*x*_ diblock copolymers with higher *x* values. TEM analysis confirmed a range of copolymer morphologies; including spherical micelles and worms (see Fig. S6 in ESI†). However, targeting PNMEP DPs greater than 250 merely led to macroscopic precipitation, hence vesicles could not be accessed under these conditions. A detailed phase diagram was constructed to aid the reproducible targeting of PSMA_14_–PNMEP_*x*_ copolymer morphologies (see Fig. 5). In particular, the effect of varying the PNMEP DP between 50 and 250 was examined for PISA syntheses conducted at 10–30% w/w solids. When PSMA_14_–PNMEP_*x*_ diblock copolymers were prepared at 10% w/w solids, then a spherical morphology was always obtained, regardless of the *x* value. At 15% w/w solids, spheres were observed for *x* values up to 173, whereas *x* = 90 is the upper limit DP for the sphere phase prepared at 30% w/w solids. These additional observations support the hypothesis that the spheres produced at lower concentrations represent a kinetically-trapped (rather than equilibrium) morphology when targeting higher PNMEP DPs.

A high proportion of the phase space shown in Fig. 5 represents mixed phases where two or more morphologies co-exist. The ‘pure’ worm phase is defined as more than 95% of nano-objects analysed by TEM being classified as worms. This highly anisotropic morphology occupies relatively narrow phase space, which is consistent with observations made by Fielding and co-workers for related RAFT dispersion polymerisation syntheses conducted in *n*-alkanes.^13,33,34^ Both Fielding *et al.* and Pei *et al.* have shown that such block copolymer worms form thermo-responsive gels, which undergo reversible degelation on heating *via* a worm-to-sphere transition.^34–36^ Fielding *et al.* explained this phenomenon in terms of surface plasticisation of the core-forming PBzMA block by the hot *n*-alkane solvent, since this lowers the effective packing parameter for the block copolymer chains.^34^ In contrast, the PSMA_14_–PNMEP_198_ worms formed in the present study do not exhibit such thermo-responsive behaviour. Presumably, this is simply because *n*-dodecane is always a very poor solvent for the highly polar PNMEP block, even at temperatures of up to 150 °C.

For other PISA formulations reported in the literature^23,45^ vesicles are typically formed at high solids when targeting relatively high core-forming block DPs. However, in the present work vesicles are produced at and above 27.5% w/w solids only when targeting PNMEP DPs of 200 or below. This is because longer core-forming blocks lead to colloidally unstable dispersions and macroscopic precipitation. Similar observations were made by Warren *et al.* for a phase diagram constructed for a poly(ethylene glycol)–poly(2-hydroxypropyl methacrylate) PISA formulation.^25^ TEM analysis of diluted dispersions of PSMA_14_–PNMEP_≥250_ nano-objects prepared at or above 25% solids confirm the presence of large vesicular aggregates (see Fig. S7†).

**Fig. 6:**
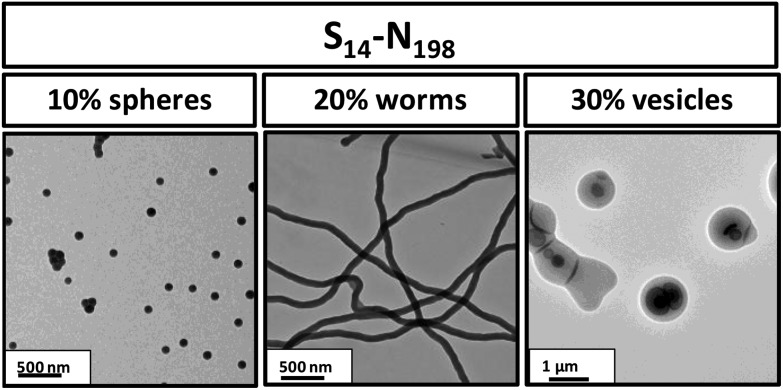
TEM images obtained for PSMA_14_–PNMEP_198_ diblock copolymer nano-objects prepared at 10, 20 or 30% w/w solids confirming the formation of well-defined spheres, highly anisotropic worms and polydisperse vesicles, respectively (N.B. for brevity ‘S’ denotes PSMA and ‘N’ denotes PNMEP).

The PSMA_14_–PNMEP_198_ composition is particularly interesting, since varying the copolymer concentration yields the full range of morphologies (spheres, worms and vesicles). Thus a near-monodisperse spherical morphology is observed at 10% w/w solids, whereas worms (approximate worm width = 100 nm, but highly polydisperse in worm contour length) are produced at 20% w/w solids and a vesicle phase comprising mainly oligolamellar vesicles^25^ is formed at 30% w/w solids (Fig. 6). This example nicely illustrates the concentration-dependent morphologies that can be obtained *via* such PISA syntheses.

### Pickering emulsifier studies

**Scheme 2:**
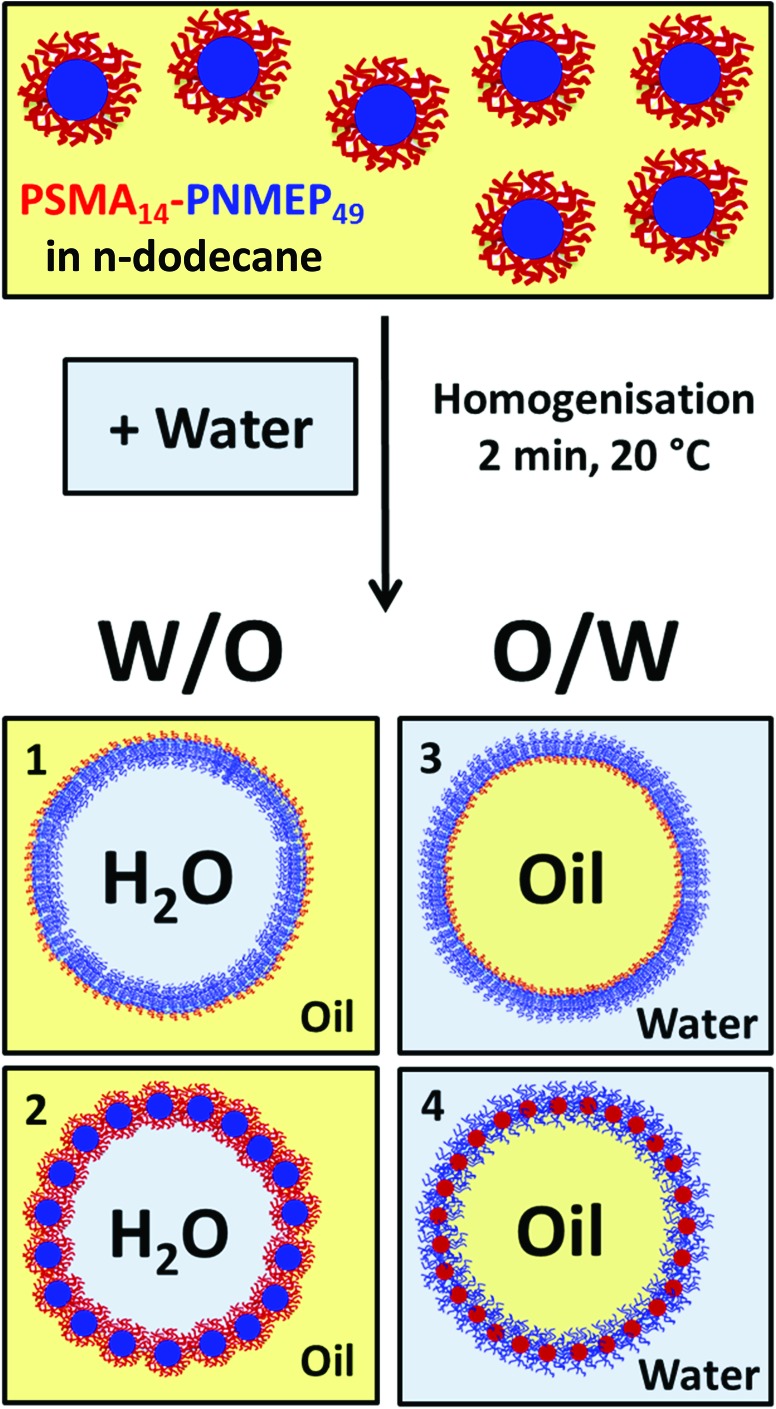
Schematic representation of the four possible types of emulsions which could form as a result of homogenising the PSMA_14_–PNMEP_49_ nanoparticles prepared in *n*-dodecane with water. In scenarios 1 and 3, the nanoparticles dissociate to produce amphiphilic diblock copolymer chains that act as a polymeric surfactant stabiliser, producing either water-in-oil or oil-in-water emulsions, respectively. In scenario 2, the hydrophobic nanoparticles are retained intact and adsorb at the oil/water interface to form water-in-oil Pickering emulsions. In scenario 4, morphological inversion occurs to form hydrophilic nanoparticles that stabilise oil-in-water Pickering emulsions.

A 10 g batch of 25 nm diameter PSMA_14_–PNMEP_49_ spheres was prepared at 10% w/w solids in *n*-dodecane for evaluation as an emulsifier. In principle, homogenisation of these *n*-dodecane nanoparticle dispersions with water could lead to four types of emulsions (Scheme 2). Scenarios 1 and 3 are expected if the nanoparticles became unstable under the homogenisation conditions and broke up to form individual diblock copolymer chains that act as a polymeric surfactant. Such *in situ* dissociation has been recently reported by Thompson and co-workers for PGMA–PHPMA spheres in water.^46^ Thus, a water-in-oil emulsion is expected if the hydrophobic PSMA block acts as the stabiliser, as indicated in scenario 1. Alternatively, according to scenario 3, an oil-in-water emulsion should be formed if the (longer) hydrophilic PNMEP acts as the stabiliser. However, based on further studies performed by Thompson *et al.* using PLMA–PBzMA worms or spheres,^47,48^ the PSMA_14_–PNMEP_49_ spheres may simply remain intact and stabilise a water-in-oil Pickering emulsion (see scenario 2). Finally, scenario 4 depicts possible inversion of the original hydrophobic PSMA_14_–PNMEP_49_ nanoparticles to form hydrophilic PNMEP_49_–PSMA_14_ spheres, which could then stabilise an oil-in-water Pickering emulsion.

**Fig. 7:**
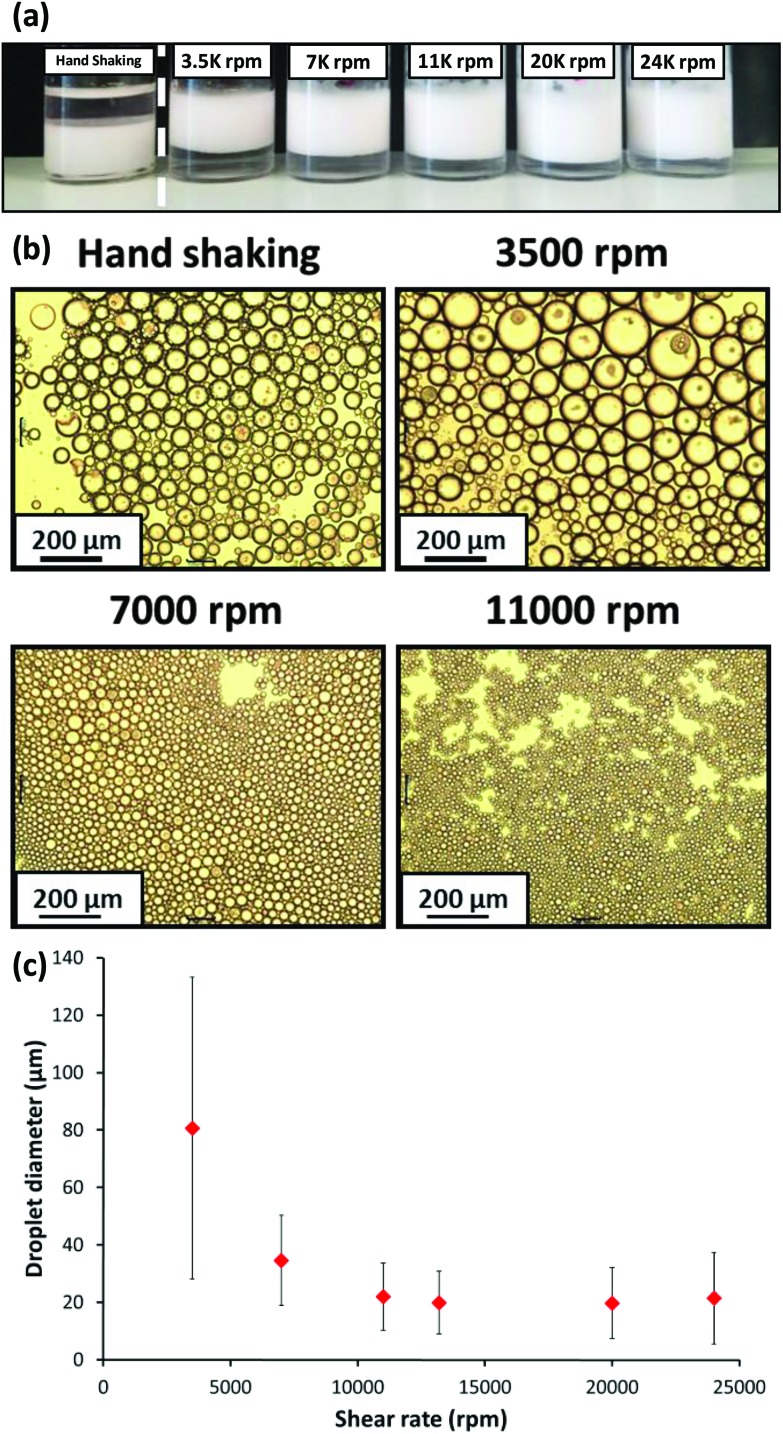
(a) Digital photographs obtained for the Pickering emulsions prepared using 1.0% w/w PSMA_14_–PNMEP_49_ nanoparticles at various shear rates. Oil-in-water emulsions are formed in all cases, except when hand-shaking is used; this latter approach results in a water-in-oil emulsion instead. (b) Optical microscopy images recorded for the droplets prepared *via* hand-shaking, or *via* homogenisation at 3500 rpm, 7000 rpm or 11 000 rpm (scale bar = 200 μm), (c) shear rate dependence for the mean droplet diameter (as determined by laser diffraction) for emulsions prepared using PSMA_14_–PNMEP_49_ spherical nanoparticles as the sole emulsifier. The error bars represent the standard deviation of each mean volume-average droplet diameter, rather than the experimental error.

Initial studies of the effect of shear rate on emulsion formation were performed using a fixed 1.0% w/w concentration of PSMA_14_–PNMEP_49_ nanoparticles. Emulsions were formed by homogenisation of a 50 : 50 v/v water/*n*-dodecane mixture at 3500 to 24 000 rpm for 2 min at 20 °C, with one additional emulsification experiment being conducted *via* hand-shaking for 2 min. Fig. 7a shows digital photographs of the resulting emulsions. The emulsion formed by hand-shaking resulted in a water-in-oil emulsion as expected, but surprisingly all other emulsions prepared at higher shear rates resulted in oil-in-water emulsions. However, at this point it was not known whether the PSMA_14_–PNMEP_49_ emulsifier was present in the form of nanoparticles or individual copolymer chains.

All emulsions were imaged by optical microscopy and selected emulsions prepared at various shear rates are shown in Fig. 7b. The effect of the shear rate on the mean droplet diameter is evident: larger droplets are formed at 3500 rpm compared to those produced at either 7000 rpm or 11 000 rpm. Laser diffraction was utilised to measure the mean diameter of the oil-in-water emulsion droplets (see Fig. 7c). A gradual reduction in mean droplet diameter with increasing shear rate was observed: ∼80 μm droplets were formed at 3500 rpm, whereas ∼20 μm droplets were obtained at shear rates above 11 000 rpm. Thompson and co-workers reported similar observations for water droplets stabilised by PLMA–PBzMA worms prepared in *n*-dodecane.^47^

**Fig. 8:**
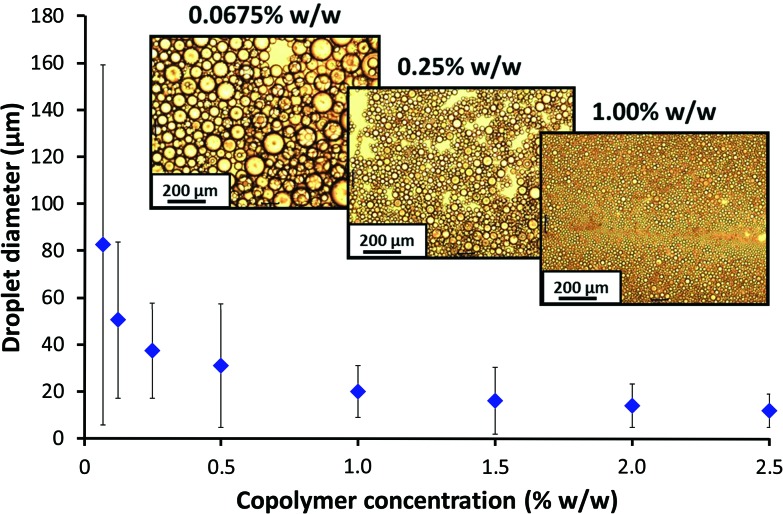
Concentration dependence of the mean volume-average droplet diameter (as determined by laser diffraction) for oil-in-water Pickering emulsions prepared at a constant shear rate of 13 200 rpm using PSMA_14_–PNMEP_49_ spheres. The error bars shown represent the standard deviation of each mean volume-average droplet diameter, rather than the experimental error. Inset: optical microscopy images of the droplets prepared at 0.0675, 0.25 and 1.00% w/w. Scale bar = 200 μm.

DLS studies were undertaken to investigate the effect of the high shear emulsification conditions on the stability of the PSMA_14_–PNMEP_49_ nanoparticles. Prior to homogenisation, colloidally stable low-polydispersity nanoparticles with an intensity-average diameter of 25 nm were observed (Fig. S8a in ESI†). After homogenisation of a 1.0% w/w nanoparticle dispersion in *n*-dodecane (*i.e.* in the absence of any added water) at 13 200 rpm for 2 min, highly polydisperse particles of 732 nm diameter were obtained. Moreover, the count rate was reduced by a factor of more than three, from 2111 kcps to 604 kcps. This suggests that the original spherical nanoparticles are unstable when subjected to high shear and undergo (at least partial) dissociation. In principle, this could potentially result in scenario 3 (Scheme 2) in which the highly amphiphilic diblock copolymer chains may act as a polymeric surfactant. To examine this hypothesis, the copolymer concentration of PSMA_14_–PNMEP_49_ spheres was varied from 0.0675% w/w to 2.50% w/w and homogenised with an equal volume of water at a fixed shear rate of 13 200 rpm to produce a series of oil-in-water emulsions. The emulsion droplet size distributions were analysed by laser diffraction, see Fig. 8. Clearly, there is a strong concentration dependence: droplets of more than 50 μm are formed at low PSMA_14_–PNMEP_49_ concentrations whereas approximately 10 μm droplets are obtained at the highest copolymer concentration. These observations are consistent with the corresponding optical microscopy images (Fig. 8, see inset). This indicates that the copolymer actually absorbs in the form of nanoparticles, rather than individual chains. This interpretation is supported by TEM studies, which confirm the presence of spherical particles adsorbed at the surface of a dried emulsion droplet (see Fig. S9 in the ESI†). Moreover, since oil-in-water emulsions are obtained rather than water-in-oil emulsions, this suggests that *in situ* inversion of the initial hydrophobic PSMA_14_–PNMEP_49_ spheres to form hydrophilic PNMEP_49_–PSMA_14_ spheres occurs, see scenario 4 in Scheme 2. This result was completely unexpected and warrants further investigation.

To further analyse the water-in-oil emulsion system obtained by hand-shaking, a series of such emulsions were prepared by hand-shaking with equal volumes of water using copolymer concentrations of 0.125% to 1.50% w/w. In each case, water-in-oil emulsions were obtained, as judged by optical microscopy (see ESI, Fig. S10†). These images suggest some concentration dependence for the mean droplet diameter but unfortunately these water-in-oil emulsions proved to be insufficiently stable to enable laser diffraction analysis. Instead, mean droplet diameters were estimated by sizing a minimum of 100 droplets per emulsion (see ESI, Fig. S11†). As expected, a concentration-dependent mean droplet diameter was observed, which suggests that nanoparticles, rather than copolymer chains, are adsorbed at the oil/water interface when homogenisation was performed at very low shear rates (*i.e.* hand-shaking). DLS analysis of the *n*-dodecane supernatant after sedimentation of the relatively dense water droplets supported this interpretation. An intensity-average diameter of 28 nm (polydispersity = 0.03) was observed (see ESI, Fig. S8b†), which is very similar to that of the original nanoparticles (intensity-average diameter = 25 nm; polydispersity = 0.07). These observations, taken together with the concentration-dependent droplet size indicated by optical microscopy studies, suggests that PSMA_14_–PNMEP_49_ spherical nanoparticles stabilise water-in-oil Pickering emulsions, see scenario 2 in Scheme 2.

Finally, the effect of varying the volume fraction of the aqueous phase was studied. Three Pickering emulsions were prepared using 0.50% w/w PSMA_14_–PNMEP_49_ nanoparticles utilising 25%, 50% or 75% water relative to the volume of nanoparticle dispersion in *n*-dodecane, with homogenisation being conducted at a constant shear rate of 13 200 rpm. Using 75% or 50% water resulted in an oil-in-water emulsion. However, using a water volume fraction of 25% led to the formation of a water-in-oil emulsion. Digital photographs of these three emulsions and their corresponding optical microscopy images are shown in Fig. S12.† These observations indicate that PSMA_14_–PNMEP_49_ spherical nanoparticles enable the preparation of water-in-oil emulsions *via* two methods: either using very low shear (hand-shaking) or by using a 25% water/75% *n*-dodecane formulation in order to prevent nanoparticle inversion.

## Conclusions

The RAFT dispersion polymerisation of a highly polar monomer, NMEP, has been conducted in *n*-dodecane using a PSMA_14_ macro-CTA to produce a series of diblock copolymer nanoparticles *via* PISA. In all cases, high conversions (≥95%) were achieved within 2 h at 90 °C. Kinetic studies for a PSMA_14_–PNMEP_100_ formulation indicated more than 99% conversion within 50 min, which is much faster than the rate of polymerisation of a non-polar monomer (benzyl methacrylate) under precisely the same conditions. GPC analysis confirmed a linear evolution in *M*_n_ when targeting higher PNMEP DPs, as expected. However, significantly broader molecular weight distributions (*M*_w_/*M*_n_ > 1.50) were observed when targeting PNMEP DPs above approximately 150. This is attributed to a dimethacrylate impurity in the NMEP monomer or perhaps chain transfer to PNMEP. TEM analysis of the PSMA_14_–PNMEP_*x*_ diblock copolymer nanoparticles prepared at 10% w/w solids indicated an exclusively spherical morphology. Both DLS and TEM studies indicated that remarkably large uniform spheres of 462 nm diameter could be obtained when targeting *x* = 1000. As far as we are aware, these are the largest spheres ever reported for PISA formulations. Conducting PISA syntheses at either 15.0 or 17.5% w/w solids enabled PSMA_14_–PNMEP_198_ worms to be obtained. These worms formed free-standing gels, but do not appear to exhibit thermo-responsive behaviour. Construction of a phase diagram enabled reproducible targeting of pure spherical micelles, worms or vesicles. PSMA_14_–PNMEP_49_ spheres were evaluated as putative Pickering emulsifiers. Water-in-oil emulsions were obtained in hand-shaking (low shear) experiments, as expected for such hydrophobic particles. However, oil-in-water emulsions were unexpectedly obtained when emulsification was conducted under high shear. This is attributed to *in situ* inversion to produce hydrophilic PNMEP_49_–PSMA_14_ spheres. Thus the same diblock copolymer spheres can form two types of Pickering emulsion depending on the emulsification conditions.
