# Supplementary material for: Synthesis, characterisation and Pickering emulsifier performance of poly(stearyl methacrylate)–poly(N-2-(methacryloyloxy)ethyl pyrrolidone) diblock copolymer nano-objects via RAFT dispersion polymerisation in n-dodecane
Source: Polym Chem. 2016 Feb 18;7(10):1882–91. doi: 10.1039/c6py00138f (PMC5361141; doi:10.1039/c6py00138f)
Supplement: Supplementary file 1 [file PY-007-C6PY00138F-s001.pdf]

Electronic Supplementary Material (ESI) for Polymer Chemistry.
This journal is © The Royal Society of Chemistry 2016

Supporting Information for:

Synthesis, characterisation and Pickering emulsifier performance of poly(stearyl methacrylate)-poly(*N*-2-(methacryloyloxy)ethyl pyrrolidone) diblock copolymer nano-objects via RAFT dispersion polymerisation in *n*-dodecane

Victoria J. Cunningham and Steven P. Armes*

Department of Chemistry, University of Sheffield,
Brook Hill, Sheffield, South Yorkshire, S3 7HF, UK.

Osama M. Musa

Ashland Specialty Ingredients, 1005 US 202/206,
Bridgewater, NJ 08807, USA.

Synthesis of $PSMA_{14}$-$PBzMA_{95}$ via RAFT dispersion polymerisation of SMA

$PSMA_{14}$ macro-CTA (0.2201 g), BzMA (0.7728 g, 4.386 mmol), T21s (2.37 mg, 26 µmol; dissolved at 10% v/v in *n*-dodecane; CTA/T21s molar ratio = 4.0) were dissolved in *n*-dodecane (4.13.9713 g, 10% w/w) in a 25 ml round-bottomed flask. The reaction mixture was sealed and purged with nitrogen for 30 min, prior to immersion in an oil bath set at 90 °C. Aliquots were taken every 10 min for the first hour and analysed by ^{1}H NMR analysis. A final conversion of 95% was achieved after 6 h. The resulting copolymer was analysed using 3:1 chloroform/methanol GPC (M_n = 23,800 g mol^{-1}, M_w/M_n = 1.10 vs. PMMA standards). DLS studies on a 0.20% w/w copolymer dispersion indicated an intensity-average particle diameter of 181 nm (DLS polydispersity, PDI = 0.260). TEM studies confirmed a worm-like morphology at 95 % conversion.

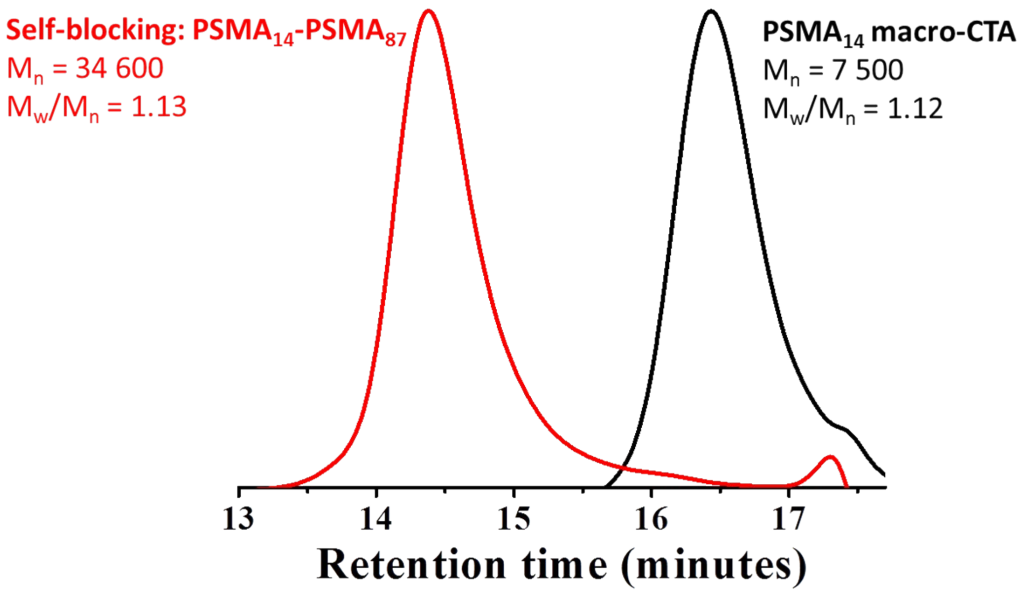

Figure S1. 3:1 Chloroform/methanol GPC curves obtained for the initial $PSMA_{14}$ macro-CTA and the corresponding $PSMA_{14}$-$PSMA_{87}$ after a 'self-blocking' chain extension experiment at 90 °C.

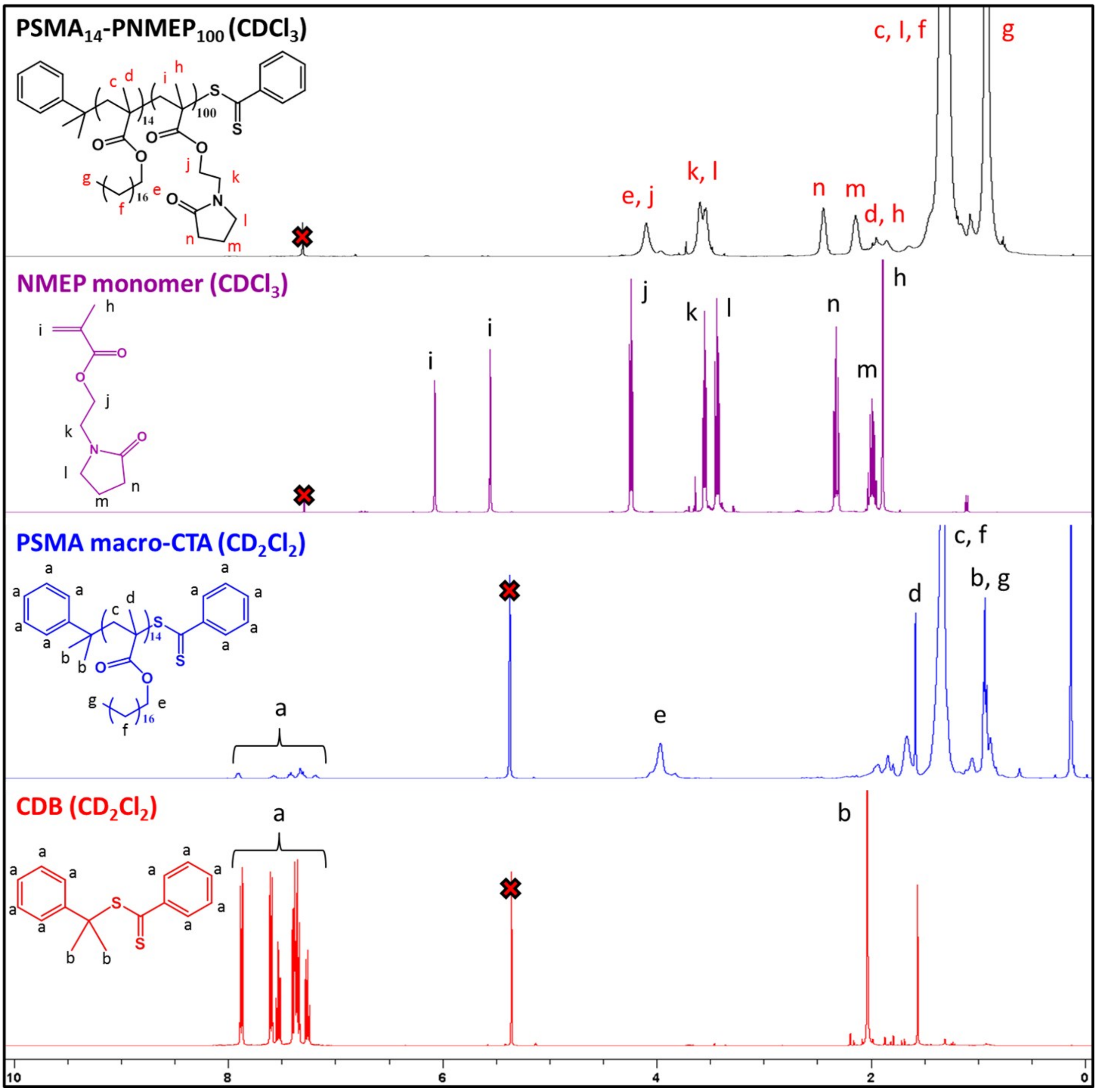

Figure S2. Assigned ^{1}H NMR spectra obtained for the CDB RAFT agent, a $PSMA_{14}$ macro-CTA, the NMEP monomer and a $PSMA_{14}$-$PNMEP_{100}$ diblock copolymer.

.

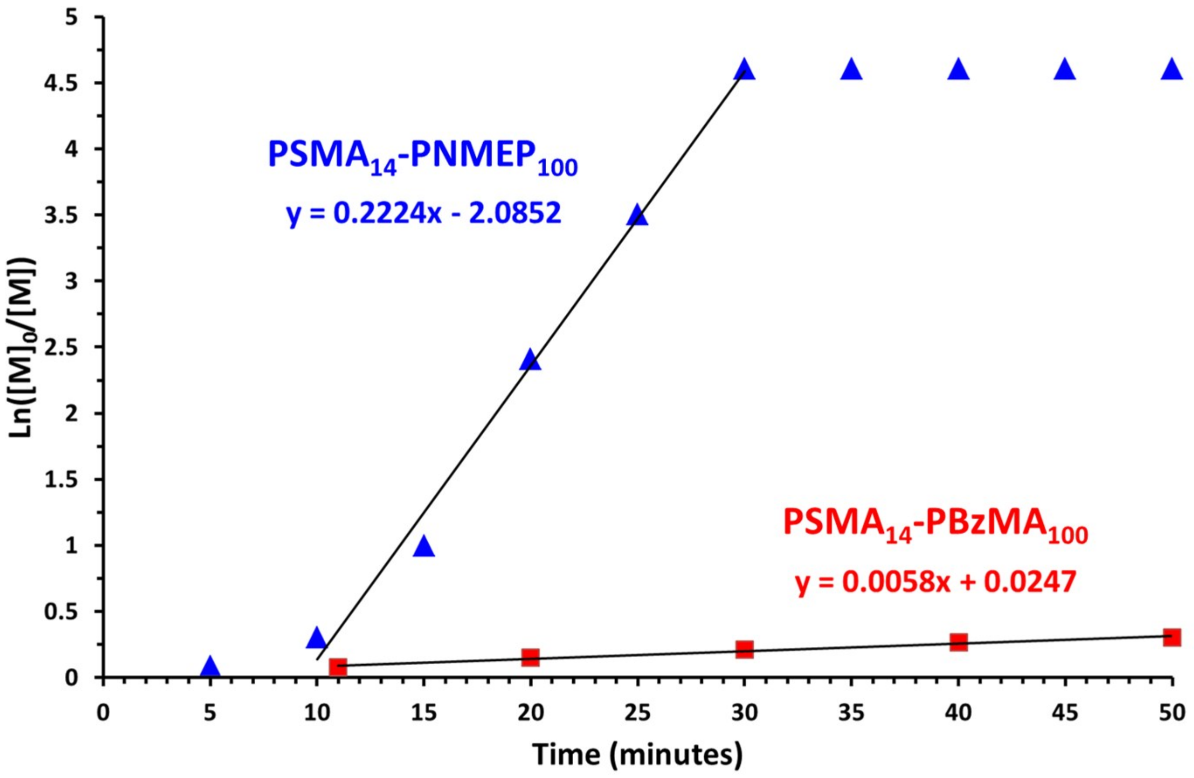

Figure S3. A plot of Ln($[M]_0/[M]$) against time for the kinetics of RAFT dispersion polymerisation of either NMEP or BzMA at 90°C when targeting either $PSMA_{14}$-$PNMEP_{100}$ (blue) or $PSMA_{14}$-$PBzMA_{100}$ (red), respectively. A short induction period is observed for the $PSMA_{14}$-$PNMEP_{100}$ formulation, but then the rate of this NMEP polymerisation proceeds with an apparent pseudo-first order rate constant that is 38 times greater than that of the BzMA polymerisation when targeting $PSMA_{14}$-$PBzMA_{100}$.

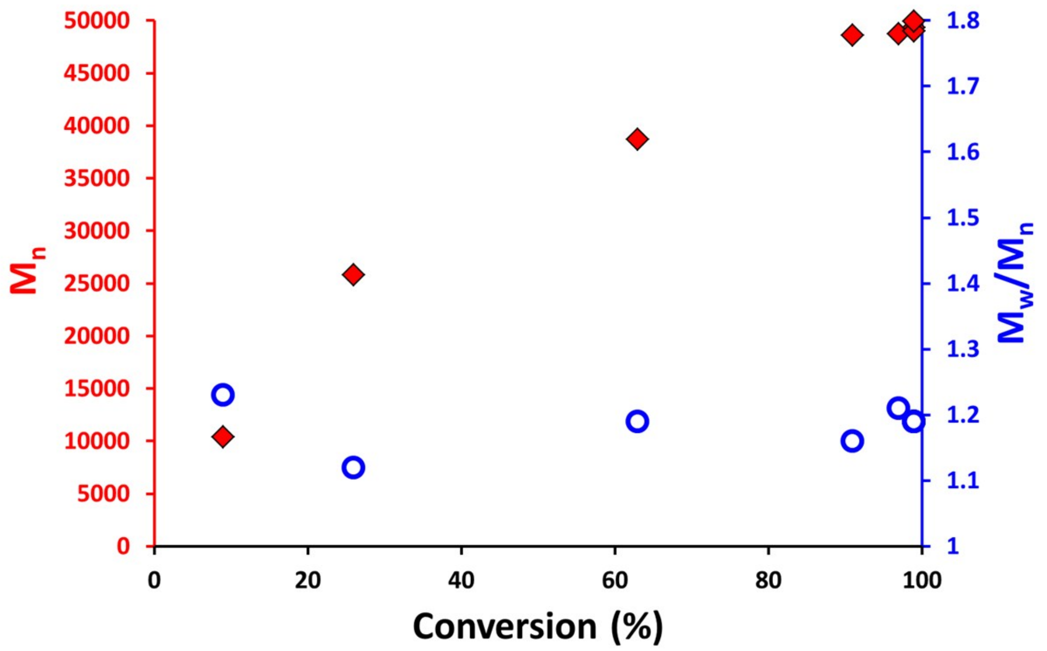

Figure S4. 3:1 Chloroform/methanol GPC data (versus a series of poly(methyl methacrylate) standards) obtained for the kinetics of polymerisation of NMEP when targeting $PSMA_{14}$-$PNMEP_{100}$

Table S1. Conversions, Molecular Weights (M_n), Polydispersities (M_w/M_n), DLS and TEM diameters obtained for $PSMA_{14}$-$PNMEP_x$ (S_{14}-N_x) Diblock Copolymer Nanoparticles prepared at 20% w/w Solids and the corresponding $PSMA_{14}$ Macro-CTA prepared at 40 % w/w Solids.

	Diblock Composition	Conversion[a] (%)	Solids content (% w/w)	3:1 v/v% chloroform methanol GPC		Particle diameter	Particle morphology
				M_n [b] (kg mol^{-1})	M_w/M_n [b]	DLS[c] (nm)	
S1	S_{14}	80	40	7.5	1.12	N/A	-
S2	S_{14}-N_{49}	99	20	29.7	1.15	26 (0.049)	Spheres
S3	S_{14}-N_{79}	99	20	44.5	1.22	36 (0.055)	Spheres
S4	S_{14}-N_{99}	99	20	52.7	1.27	39 (0.054)	Spheres
S5	S_{14}-N_{114}	95	20	63.3	1.34	48 (0.054)	Spheres
S6	S_{14}-N_{149}	99	20	84.3	1.59	151 (0.093)	Mixed
S7	S_{14}-N_{173}	99	20	85.0	1.48	1174 (0.324)	Mixed
S8	S_{14}-N_{190}	>99	20	89.8	1.75	824 (0.305)	Mixed
S9	S_{14}-N_{198}	99	20	99.4	2.01	722 (0.348)	Worms
S10	S_{14}-N_{209}	99	20	105.8	1.79	729 (0.461)	Mixed
S11	S_{14}-N_{221}	98	20	111.5	1.94	1370 (0.302)	Mixed
S12	S_{14}-N_{248}	99	20	121.9	2.04	1169 (0.115)	Mixed

a. Monomer conversion determined by ^{1}H NMR spectroscopy in $CDCl_3$.
b. Determined by 3:1 v/v chloroform/methanol GPC against poly(methyl methacrylate) calibration standards using a refractive index detector.
c. The numbers in brackets refer to the DLS polydispersity

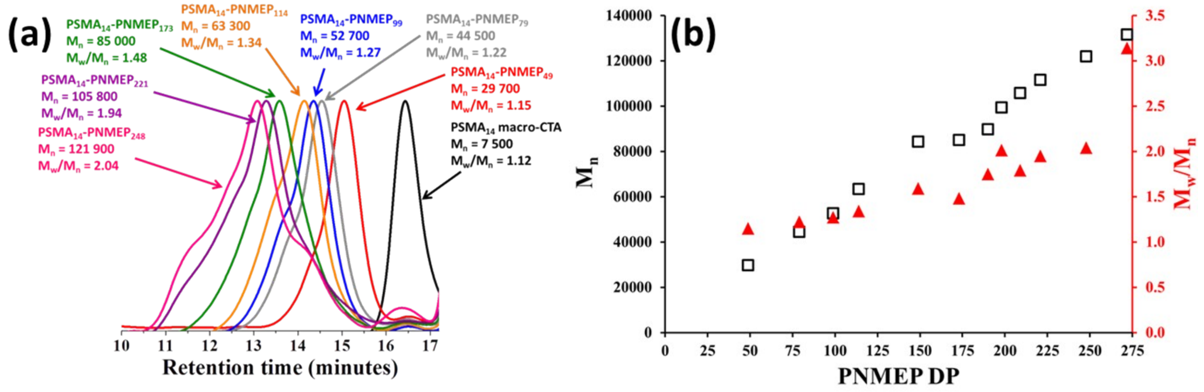

Figure S5. (a) 3:1 Chloroform/methanol GPC curves obtained for a selection of $PSMA_{14}$-$PNMEP_x$ diblock copolymer nanoparticles prepared at 20% w/w solids via RAFT dispersion polymerisation of NMEP at 90 °C. (b) A plot of M_n (black axis) and M_w/M_n (red axis) against PNMEP DP for the same series of $PSMA_{14}$-$PNMEP_x$ diblock copolymer nanoparticles.

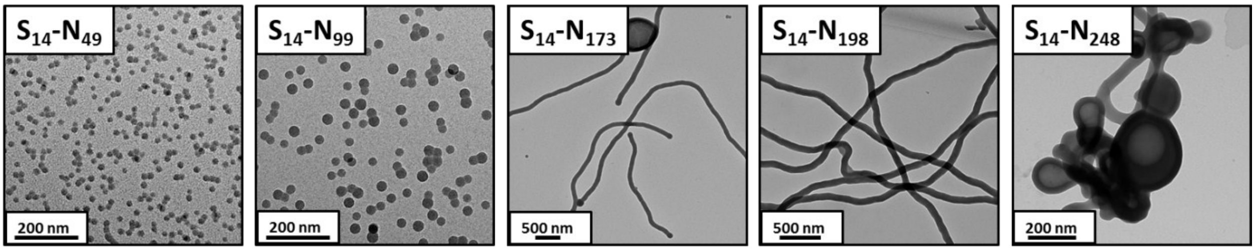

Figure S6. Representative TEM images obtained for $PSMA_{14}$-$PNMEP_x$ diblock copolymer nano-objects prepared via RAFT dispersion polymerisation of NMEP at 20% w/w solids.

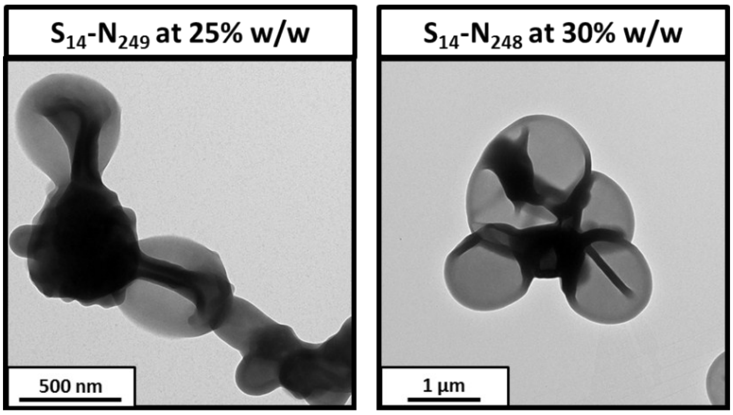

Figure S7. Representative TEM images recorded for the unstable precipitate phase obtained for $PSMA_{14}$-$PNMEP_x$ syntheses conducted at either 25% or 30% solids when targeting a PNMEP DP of 250.

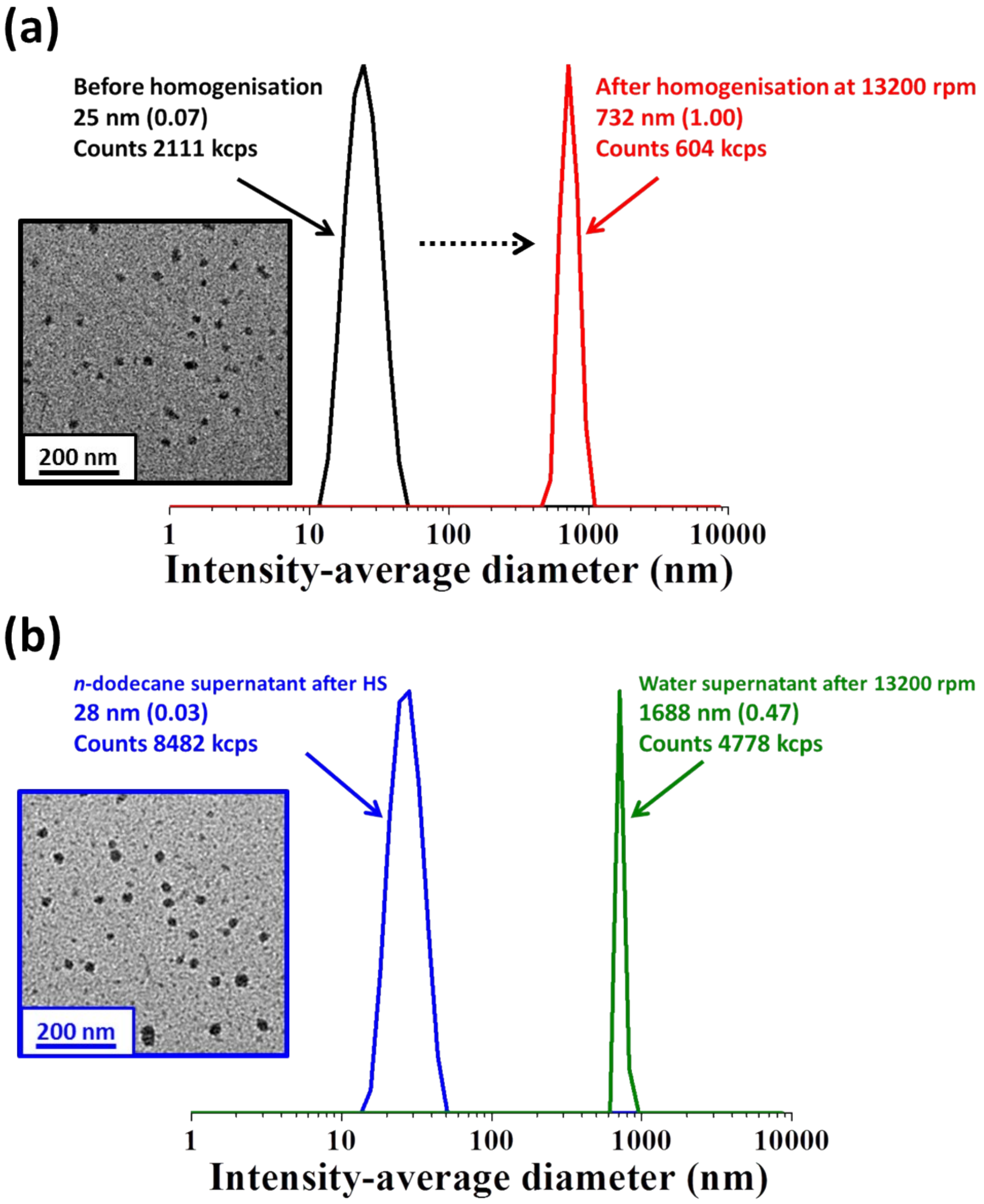

Figure S8. (a) DLS particle size distributions obtained at 25°C for $PSMA_{14}$-$PNMEP_{49}$ nanoparticles before (black) and after (red) homogenisation at 13,200 rpm at 20°C. High shear homogenisation causes the initial 25 nm spherical nanoparticles to break up, resulting in a relatively low count rate and a very high polydispersity. Inset: TEM image before homgenisation. (b) DLS particle size distributions obtained for the *supernatants* (after gravitational sedimentation of the aqueous droplet phase on standing at 20°C overnight) of water-in-oil emulsions prepared using 1.0% w/w $PSMA_{14}$-$PNMEP_{49}$ nanoparticles. The blue trace shows the DLS size distribution (and corresponding TEM image) obtained for the *n*-dodecane *supernatant* of the water-in-oil emulsion prepared by hand-shaking, confirming that these nanoparticles are stable to homogenisation at *low* shear (hand-shaking). The green trace shows the *aqueous* supernatant of the oil-in-water emulsion obtained after preparation via homogenisation at 13,200 rpm.

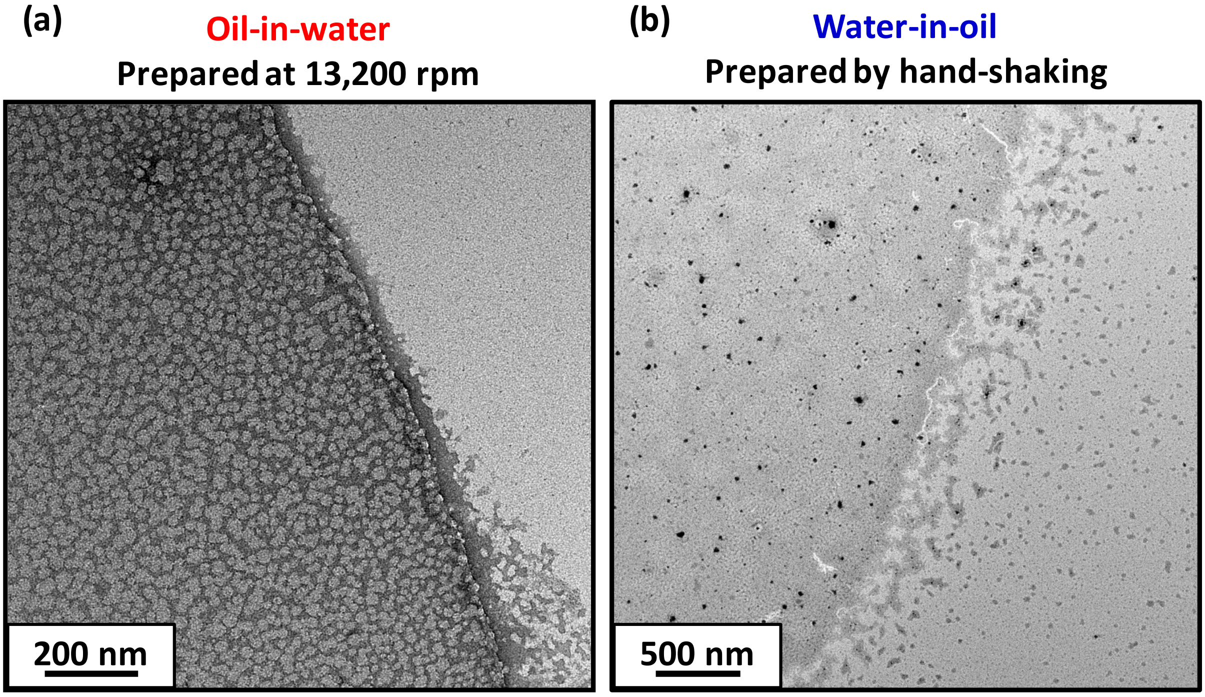

Figure S9. Transmission electron microscopy of Pickering emulsion droplets prepared using $PSMA_{14}$-$PNMEP_{49}$; (a) an oil-in-water droplet prepared at 13,200 rpm and stained using uranyl formate and (b) a water-in-oil droplet prepared by hand-shaking and stained using ruthenium(VIII) oxide. Both show the presence of spherical nanoparticles absorbing at the oil-water interface.

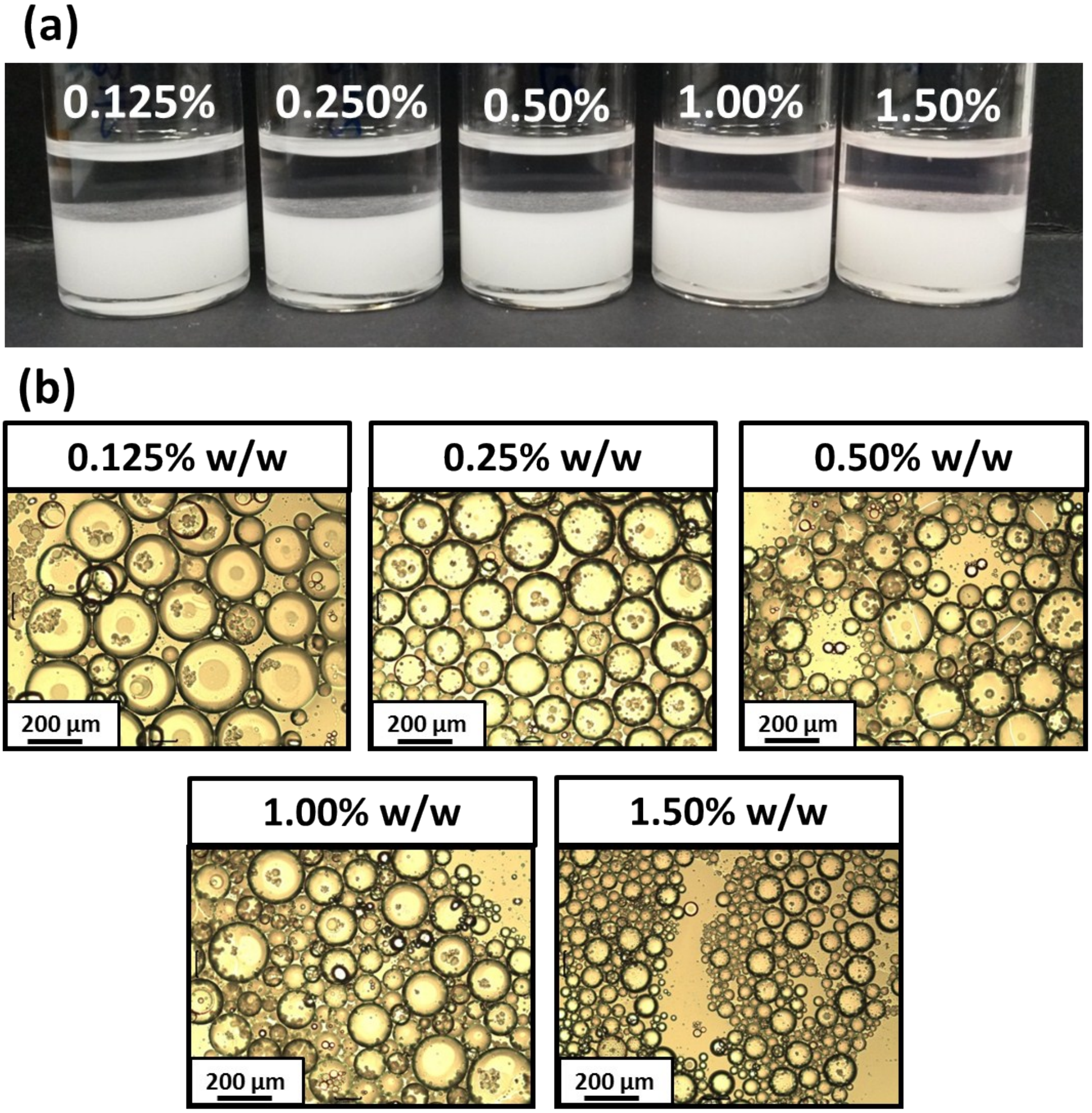

Figure S10. Water-in-oil emulsions prepared by hand-shaking of 50:50v/v mixtures of *n*-dodecane and water using either 0.125%, 0.25% 0.50%, 1.00% or 1.50% w/w $PSMA_{14}$-$PNMEP_{49}$ nanoparticles: (a) digital photograph of the resulting five emulsions and (b) corresponding optical microscopy images.

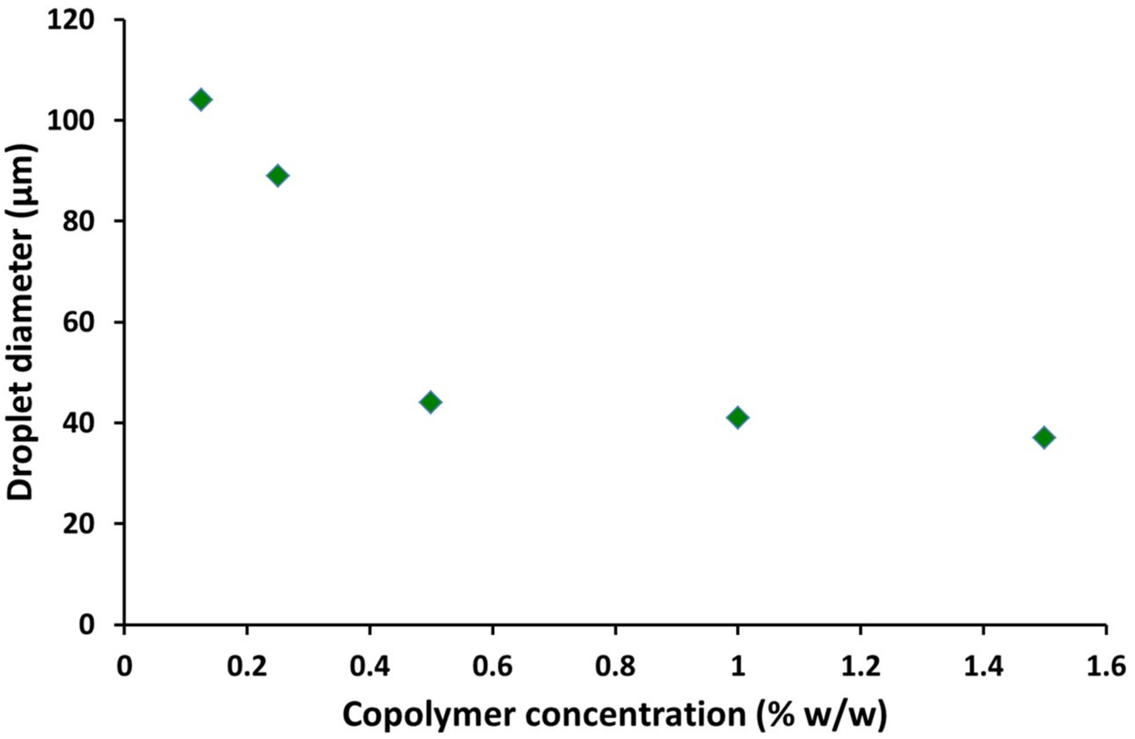

Figure S11. Concentration dependence of the mean aqueous droplet diameter obtained for the water-in-oil emulsions prepared by hand-shaking using either 0.125%, 0.25% 0.50%, 1.00% or 1.50% w/w $PSMA_{14}$-$PNMEP_{49}$ nanoparticles. Mean droplet diameters were estimated from optical microscopy images by analysing at least 100 droplets.

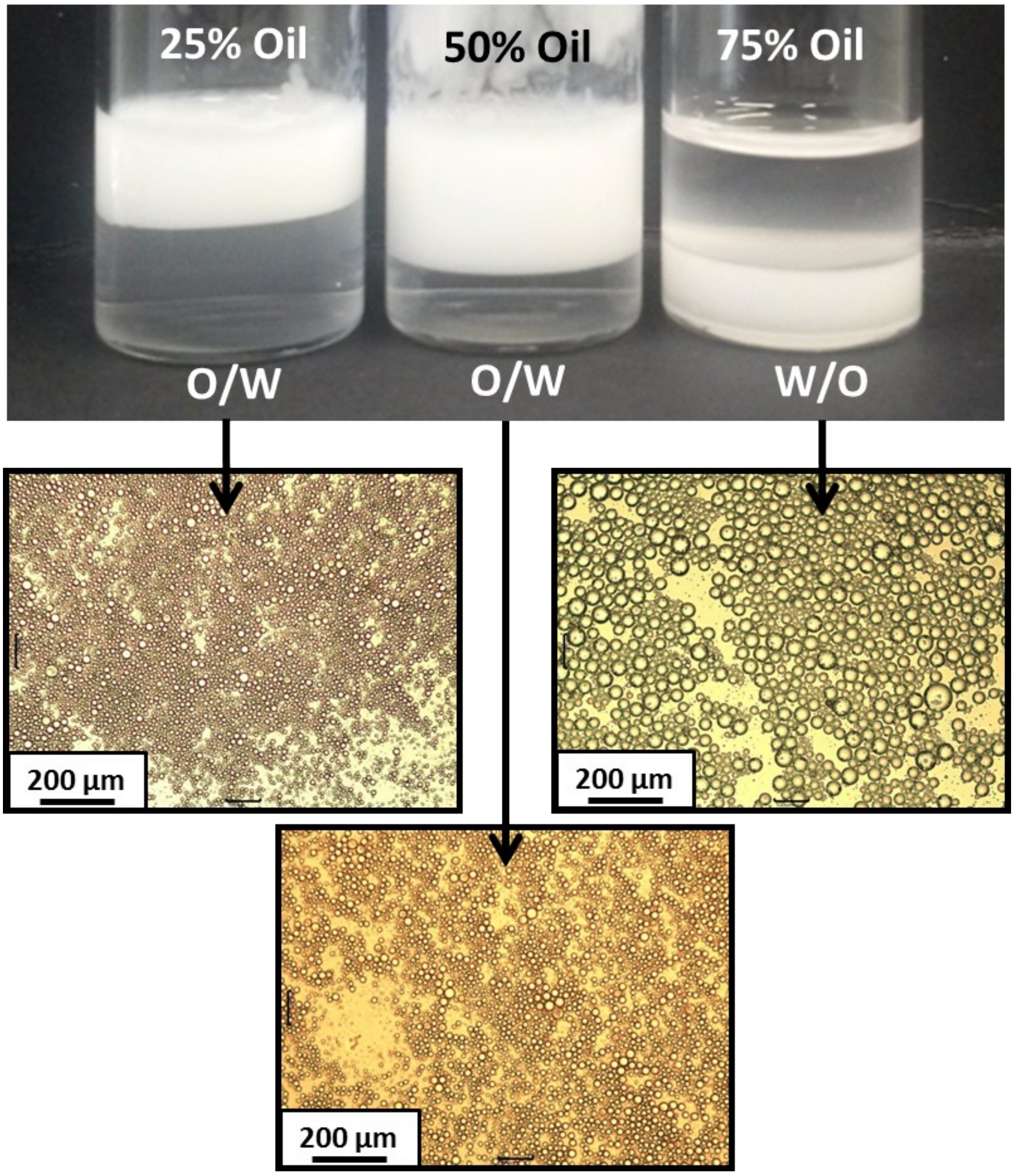

Figure S12. Digital photographs and corresponding optical microscopy images obtained for $PSMA_{14}$-$PNMEP_{49}$ emulsions formed using water volume fractions 0.25, 0.50 or 0.75 relative to *n*-dodecane. Emulsions were prepared using 0.5% w/w $PSMA_{14}$-$PNMEP_{49}$nanoparticles at 13,200 rpm. The latter water volume fractions resulted in the formation of *oil-in-water* emulsions, whereas the lowest water volume fraction produced a *water-in-oil* emulsion.
